# Incentive mechanism for sharing and using EHR in medical consortiums based on performance evaluation

**DOI:** 10.3389/fpubh.2023.1324228

**Published:** 2024-01-05

**Authors:** Sheng Hu Tian, Rong Jiang, Yu Chen

**Affiliations:** ^1^Business School, Yunnan University of Finance and Economics, Kunming, China; ^2^Yunnan Key Laboratory of Service Computing, Yunnan University of Finance and Economics, Kunming, China; ^3^School of Management and Economics, Kunming University of Science and Technology, Kunming, China

**Keywords:** electronic health records (EHR), medical consortiums, incentive mechanism, performance appraisal (PA), health information

## Abstract

**Background:**

The construction of medical consortiums not only promotes active cooperation among hospitals, but also further intensifies active competition among them. The shared use of electronic health records (EHR) breaks the original pattern of benefit distribution among hospitals.

**Objective:**

The purpose of this paper is to establish an incentive mechanism for the shared use EHR, and to reveal the incentive effect and mechanism of key factors, and to put forward management suggestions for solving the real conflicts.

**Methods:**

We constructed a basic incentive model and an incentive model that introduces performance evaluation as a supervisory signal, based on analyzing the hospital cost function, the hospital benefit function, and the incentive contract function. Finally, the incentive effects of key factors before and after the introduction of performance evaluation were verified and compared using MATLAB simulation method.

**Results:**

The profit level and incentive coefficient of hospitals sharing EHR are independent of the amount of one-time government subsidies. Regardless of whether a performance evaluation supervisory signal is introduced or not, the incentive coefficients are increasing functions with respect to ρ, τ, but decreasing functions with respect to β, δ, γ. After the inclusion of supervisory signal of performance evaluation in the model, the ability of hospitals to use EHR has a higher impact effectiveness on improving both incentive effects and benefit levels. The impact of the value-added coefficient on the level of earnings is consistently greater than it would have been without the inclusion of the performance evaluation supervisory signal.

**Conclusions:**

Enhancing the capacity of hospitals to use EHR and tapping and expanding the value-added space of EHR are 2 key paths to promote sustainable shared use of EHR. Substantive performance evaluation plays an important role in stabilizing incentive effects.

## Introduction

A medical consortium is usually composed of public hospitals at different levels within a specific region, and is a consortium that provides primary care, two-way referral, emergency and slow treatment, and up and down linkage of diagnostic and treatment services. While the construction of the medical consortiums promotes active cooperation among hospitals, it also further intensifies proactive competition among hospitals. However, the shared use of EHR resources will break the original pattern of benefit distribution among hospitals. Patients who originally chose to seek medical treatment at this hospital may choose to seek medical treatment at another hospital. Which will further exacerbate the fierce competition among hospitals, thus hindering the realization of the goal of building medical consortiums (1). The integration of electronic health records (EHR) in medical consortiums is divided into two levels: the interconnection of infrastructures such as software and hardware, and the shared use of EHR resources. Interconnection is not the end, shared use is the only way to create value. To reduce the “free riding” phenomenon in the integration of EHR and promote the active sharing and use of EHR resources, it is necessary to establish an effective incentive mechanism that coordinates the positive actions of all parties.

Incentive mechanisms research is the core proposition for solving problems such as alliance cooperation, multi-agent collaboration and collaborative innovation. Incentive mechanism research is also an effective method to study the path of activating the motivation of participating subjects, which has been widely applied in the fields of supply chain management (2), online marketing (3), venture capital (4), benefit distribution (5), information sharing (6, 7), data quality (8), and innovation dynamics (9, 10). In the field of healthcare, incentive issues have always been a major focus and difficulty in practice. However, incentive mechanism research has not been paid much attention to until the beginning of this century in China, with a view to fully stimulating the enthusiasm and innovative vitality by clarifying incentive factors and revealing the mechanism of driving forces. Li and Zheng (11) studied the incentive and constraint mechanism in the coordination of the conflict between individual and collective interests in the construction of county medical communities. Xiong et al. (12) took Sanming Medical Community as the case to reveal the economic incentive mechanism in the supply of medical services. Fu (13) focuses on the incentive mechanism of medical insurance payment reform for physicians.

EHR sharing within the consortium breaks through the limitations of information sharing within individual hospitals. Its core essence is the sharing and interoperability of diagnostic and treatment information between hospitals. Cross-organizational healthcare information sharing has been recognized as a typical scientific problem for future research (14). However, most current researches mainly start from the technical perspective, exploring the system architecture and key technology solutions for information sharing between hospitals (15–17). Some scholars have also begun to pay attention to sharing risks (18), sharing willingness (19), privacy protection (20), sharing models (21), service models (22) and influencing factors (23). In recent years, research on the incentive mechanism for sharing medical and health information, with electronic health records as the core, has gradually received attention from the academic community. For example, Li et al. (24) used a game approach to analyze the incentive mechanism for sharing hospital diagnosis and treatment information. A small number of scholars, such as Jiang et al. (25), have also paid attention to and explored this topic. However, academic research on incentive mechanisms in the shared use of EHRs is far from adequate, with a small number of relevant studies and a depth of research that needs to be further improved.

In view of the above, this paper constructed a basic incentive model and an incentive model that introduces performance evaluation, respectively, based on the principal-agent theory. The model results were solved and the incentive effects ware comparatively analyzed using numerical simulation. The significance of this research is mainly reflected in three aspects: first, it reveals the incentive effect and mechanism of key factors in promoting the shared use of EHR; second, it further proves the important role of performance evaluation in the design of contractual incentive mechanism; and third, it provides decision-making reference for the practice of integrating EHR in medical consortiums.

## Methods

### Basic incentive model construction

#### Cost function of hospitals

Hospitals may need to add equipment and modify interfaces to achieve EHR sharing. In order to make EHRs directly available to other hospitals after sharing, it is necessary to integrate data and optimize the format. This inevitably requires investment in manpower and resources, which entails a certain cost. We refer to this cost as the shared cost. The amount of shared costs invested is closely related to the hospital's effort, which can be characterized by the effort level. The effort level can be expressed as $\frac{1}{2}\beta M^{2}$ (26), where β represents the cost coefficient of hospital investment, and *M* represents the hospital's earnings level (benefit level). There are also certain risks for hospitals in the EHR integration. These potential risks may include reduced revenues due to interoperability of test results, and patients loss due to convenient access to medical care. Such potential risk cost can be expressed as $\frac{1}{2}\gamma a^{2}\delta^{2}$ (27). Where γ denotes the risk avoidance intensity of the hospital, determined by the risk awareness of the hospital's decision-making level. α indicates the incentive intensity, and δ represents the uncertain impact from random factors. Therefore, the actual investment cost of the hospital is $C_{h}=\frac{1}{2}\beta M^{2}+\frac{1}{2}\gamma a^{2}\delta^{2}$. Where, δ~*N*(0, σ^2^), indicates that the greater the variance of the random disturbance variable δ, the more the potential risk of the hospital is associated with the magnitude of its effort.

#### Benefit function of hospitals

After realizing the integration of EHR, not only can it bring economic benefits to hospitals, moreover, hospitals can obtain good social benefits. The economic benefits mainly include: first, the one-time subsidy given by the government to hospitals during the EHR interconnection stage; second, the incentive subsidy given based on the performance of EHR sharing and use; and third, there is increased patient consumption, which results from the improved access efficiency and service quality due to EHR sharing. Social benefits mainly refer to good patient reputation, social image, and field status, which in turn are the foundation for improving hospital economic benefits. The potential social benefits of EHR integration include,: firstly, the public praise for reducing the financial burden of patients by reducing repeated examinations, which will attract more patients to seek medical treatment; secondly, the interconnection and interoperability of EHR can vigorously promote hierarchical diagnosis and treatment as well as upward and downward referrals, which will alleviate the heavy burden of large hospitals; and thirdly, the sharing and using EHR can enhance the accessibility of high-quality healthcare resources, and improve the overall regional healthcare conditions and environment for seeking medical treatment (28, 29). At the same time, it can strongly support hospitals to expand their existing business, such as providing Internet health consultation and health management.

The above benefits of hospitals is not only related to the profit level of the EHR (*M*), but also to the value-added coefficient of the EHR (τ), and to the coefficient of the hospital's ability to use the EHR (ρ). Therefore, the hospital's revenue can be expressed as π_*h*_(ρ, τ) = ρτM, where τ≥0 and ρ≥0. Interconnection does not add value to EHRs. The value added depends on the portion of EHR that are actually being used after sharing. Therefore, the value-added coefficient τ is actually the ratio of the number of EHR that are actually used to the number of all EHRs that are shared, and obviously 0 ≤ τ ≤ 1.

#### Incentive contract function

The government, to promote the EHR integration in medical consortiums, is bound to incentivize hospitals to actively participate in the sharing and use of EHRs. Generally, the government establishes incentive contracts either directly or by delegating to the medical consortiums. Assume that the one-time subsidy given by the government to hospitals in the EHR interconnection phase is *H*. The incentive contract function can be expressed as *S*_*h*_ = (1−α)*H*+απ_*h*_(ρ, τ), where α is the incentive coefficient, 0 ≤ α ≤ 1. The incentive coefficients not only characterize the promotion strength for EHR integration across different consortiums or geographic regions, but also can accommodate trends in the evolution of incentive strength over time. When α = 0, it means that the hospital does not bear the shared risk and does not receive the performance rewards; when α = 1, it means that the hospital bears all the shared risk and receives the full performance rewards.

#### Basic incentive model

The above analysis shows that the expected benefits of the hospital are as follows:


(1) $${\text{EV}}_{\text{h}}=(1-\alpha )H+\alpha \rho \tau M-\frac{1}{2}\beta M^{2}-\frac{1}{2}\gamma \alpha^{2}\delta^{2}$$

The expected benefits of the medical consortiums are as follows:


(2) $$\begin{array}{l} \text{E}{\text{V}}_{\text{c}}=\left(1-\alpha \right)\left(\rho \tau M-H\right) \end{array}$$

Therefore, the basic incentive model can be constructed as shown in Eq. (3):


(3) $$\{\begin{array}{c} maxE\left(V\right)=\max\left[\left(1-\alpha )(\rho \tau M-H\right)\right]\\ s.t.\left(IR\right)\;(1-\alpha )H+\alpha \rho \tau M-\frac{1}{2}\beta M^{2}-\frac{1}{2}\gamma a^{2}\delta^{2}\geq \bar{U}\\ \left(IC\right)\tau^{*}\in argmax\left[\;(1-\alpha )H+\alpha \rho \tau M-\frac{1}{2}\beta M^{2}-\frac{1}{2}\gamma a^{2}\delta^{2}\right],\forall M^{*}\in A \end{array}$$

Where, *IR* denotes the participation constraint, and *IC* represents the incentive constraint. $\bar{U}$ indicates the minimum utility before hospitals share EHR, which is the expected benefit of the hospital in the absence of incentive measures. *A* refers to the set of possible profit levels of the medical consortiums.

### Incentive model of introducing performance evaluation construction

The basic incentive model allows hospitals to learn about each other's total shared EHR. However, the effort level hospitals can put into using shared EHR cannot be directly observed. To measure the hospital's effort level more accurately, it is necessary to design a supervisory signal in the incentive mechanism. Performance evaluation is the most commonly used supervision method in reality. After introducing a supervision signal using performance evaluation as a means, the final benefit of hospitals is not only related to the benefit level from using EHR, but also to the effectiveness of performance evaluation, thereby further improving the fairness of incentive contract.

Government and hospitals constitute a principal-agent relationship. The government can only indirectly judge and measure the efforts of hospitals through performance evaluation. Because there is a cost associated with conducting performance evaluations, which can be expressed in terms of *kM*. Where *k* denotes the effectiveness coefficient of performance evaluation. Larger k means that the results of performance evaluation are more realistic. For hospitals, performance evaluation may identify deficiencies in their shared use of EHRs, which will affect the amount of performance rewards. Performance evaluation effectiveness may be affected by random factors such as policy changes, optimization of evaluation criteria, information asymmetry. These potential stochastic effects are uniformly incorporated into δ to represent. With the inclusion of performance evaluation in the model, the one-time subsidy provided by the government will be affected by the results of the performance evaluation. Drawing inspiration from the research on supervision signals in the design of incentive mechanisms (30), when adding performance evaluation supervision signals to measure the hospital's effort level, the incentive model is as follows:


(4) $$\{\begin{array}{c} maxE\left(V\right)=\max\left[(1-\alpha )(\rho \tau M-H)-kM\right]\\ s.t.\left(IR\right)\;(1-\alpha )\;H+\alpha \rho \tau M+kM-\frac{1}{2}\beta M^{2}-\frac{1}{2}\gamma (\alpha^{2}+k^{2})\delta^{2}\geq \bar{U_{0}}\\ \left(IC\right)\tau^{*}\in argmax\left[\;(1-\alpha )H+\alpha \rho \tau M+kM-\frac{1}{2}\beta M^{2}-\frac{1}{2}\gamma (a^{2}+k^{2})\delta^{2}\right],\forall M^{**}\in A \end{array}$$

Where, *IR* denotes the participation constraint, and *IC* represents the incentive constraint. $\bar{U_{0}}$ denotes the retained utility of the hospital in the event of a performance evaluation. *A* is the set of possibilities for the hospital's level of effort.

## Results and discussion

### Solution and analysis of basic incentive model

By taking the first derivative of M for IC in (3) and setting the derivative value to 0, we get


(5) $$\begin{array}{l} M=\frac{\alpha \rho \tau }{\beta } \end{array}$$

To ensure the enthusiasm of hospitals for sharing and using EHR, the benefits should not be less than the minimum utility before sharing and using EHR. Let *IR* in Eq. (3) take the equal sign and substitute (1−α)*H* into the objective function to transform the above optimization problem as follows:


(6) $$\begin{array}{l} maxE\left(V\right)=\rho \tau M-\frac{1}{2}\beta M^{2}-\frac{1}{2}\gamma \alpha^{2}\delta^{2}-\bar{U} \end{array}$$

Taking the first order derivative of α with respect to Eq. (6), we get:


(7) $$\begin{array}{l} \alpha^{{\;}^{*}}=\frac{\rho^{2}\tau^{2}}{\beta \gamma \delta^{2}+\rho^{2}\tau^{2}} \end{array}$$

Substituting α^*^ into Eq. (5), we get:


(8) $$\begin{array}{l} M^{{\;}^{*}}=\frac{\rho^{3}\tau^{3}}{\beta^{2}\gamma \delta^{2}+\beta \rho^{2}\tau^{2}} \end{array}$$

As shown in Eqs. (7) and (8), both the level of hospital profit and the incentive coefficient are independent of the amount of one-time subsidy from the government. Therefore, one-time subsidies from the government are not the driving force to share and use EHRs in the long term, and cannot generate sustainable incentives. But it does not mean that the Government's one-time subsidies should be ignored because it is a safeguarding factor. When certain conditions are met, one-time subsidy from the government can help promote the smooth integration of EHR.

By taking partial derivatives of β, γ, ρ, τ and δ in Eq. (7), respectively, the relationship of the incentive coefficient α by a parameter can be obtained as follows:


$$\begin{array}{l} \frac{\partial \alpha^{\text{*}}}{\partial \rho }=\frac{2\beta \gamma \delta^{2}\rho^{2}\tau^{2}}{{\left(\beta \gamma \delta^{2}+\rho^{2}\tau^{2}\right)}^{2}}\geq 0\\ \frac{\partial \alpha^{\text{*}}}{\partial \tau }=\frac{2\beta \delta^{2}\rho^{2}\tau }{{\left(\beta \gamma \delta^{2}+\rho^{2}\tau^{2}\right)}^{2}}\geq 0\\ \frac{\partial \alpha^{\text{*}}}{\partial \beta }=-\frac{\delta^{2}\rho^{2}\tau^{2}\gamma }{{\left(\beta \gamma \delta^{2}+\rho^{2}\tau^{2}\right)}^{2}}\leq 0\\ \frac{\partial \alpha^{\text{*}}}{\partial \gamma }=-\frac{2\beta \delta^{2}\rho^{2}\tau^{2}}{{\left(\beta \gamma \delta^{2}+\rho^{2}\tau^{2}\right)}^{2}}\leq 0\\ \frac{\partial \alpha^{\text{*}}}{\partial \delta }=-\frac{2\beta \delta \gamma \rho^{2}\tau^{2}}{{\left(\beta \gamma \delta^{2}+\rho^{2}\tau^{2}\right)}^{2}}\leq 0 \end{array}$$

The above analysis shows that α^*^ is a decreasing function with respect to β, δ and γ and an increasing function with respect to ρ and τ. As the investment cost of hospitals increases, or as the risk avoidance intensity of hospitals increases and the risks brought by random disturbances increase, the government or medical consortium must also increase the incentive intensity to achieve the incentive purpose. With the continuous growth of hospitals' ability to share and use EHR, as well as the continuous increase in the value-added coefficient of EHR, the government can gradually reduce the incentive intensity appropriately, which will also fulfill the incentive purpose.

### Solution and analysis of incentive models introducing performance evaluation

By taking the first derivative of *M* for IC in Eq. (4) and setting the derivative value to 0, we get


(9) $$\begin{array}{l} M^{{\;}^{\prime \prime }}=\;\frac{k+\alpha \rho \tau }{\beta } \end{array}$$

Let *IR* in Eq. (4) take the equal sign and substitute (1−α)*H* into the objective function to transform the above optimization problem as follows:


(10) $$\begin{array}{l} maxE\left(V\right)=\rho \tau M-\frac{1}{2}\beta M^{2}-\frac{1}{2}\gamma {\left(a^{2}+k^{2}\right)\delta }^{2}-\bar{U_{0}} \end{array}$$

By taking the partial derivatives of α and *k* in Eq. (10), respectively, and making the derivative value zero, the following results can be obtained:


(11) $$\begin{array}{l} \alpha =\frac{\rho^{2}\tau^{2}-\rho \tau k}{\beta \gamma \delta^{2}+\rho^{2}\tau^{2}} \end{array}$$

(12) $$\begin{array}{l} k=\frac{\rho \tau \left(1-\alpha \right)}{\beta \delta^{2}\gamma +1} \end{array}$$

Combining Equations (11) and (12) and solving the system of equations by the elimination method, we get:


(13) $$\begin{array}{l} \alpha^{{\;}^{**}}=\frac{\rho^{2}+\tau^{2}}{\beta \gamma \delta^{2}+\rho^{2}\tau^{2}+1} \end{array}$$

(14) $$\begin{array}{l} k^{{\;}^{**}}=\frac{\rho \tau }{\beta \gamma \delta^{2}+\rho^{2}\tau^{2}+1} \end{array}$$

Substituting α^**^ and k^**^ into Eq. (9), we obtain:


(15) $$\begin{array}{l} M^{{\;}^{**}}=\frac{\rho^{3}\tau^{3}+\rho \tau }{\beta \left(\beta \gamma \delta^{2}+\rho^{2}\tau^{2}+1\right)} \end{array}$$

The effect of several parameters on the incentive coefficient α can be obtained by taking the partial derivatives of the variables in Eq. (13) separately, as follows:


$$\begin{array}{l} \frac{\partial \alpha^{\text{**}}}{\partial \rho }=\frac{2\rho \tau^{2}\left(\beta \gamma \delta^{2}+1\right)}{{\left(\beta \gamma \delta^{2}+\rho^{2}\tau^{2}+1\right)}^{2}}\geq 0\\ \frac{\partial \alpha^{\text{**}}}{\partial \tau }=\frac{2\rho^{2}\tau \left(\beta \gamma \delta^{2}+1\right)}{{\left(\beta \gamma \delta^{2}+\rho^{2}\tau^{2}+1\right)}^{2}}\geq 0\\ \frac{\partial \alpha^{\text{**}}}{\partial \gamma }=-\frac{\beta \delta^{2}\rho^{2}\tau^{2}}{{\left(\beta \gamma \delta^{2}+\rho^{2}\tau^{2}+1\right)}^{2}}\leq 0\\ \frac{\partial \alpha^{\text{**}}}{\partial \delta }=-\frac{2\beta \delta \gamma \rho^{2}\tau^{2}}{{\left(\beta \gamma \delta^{2}+\rho^{2}\tau^{2}+1\right)}^{2}}\leq 0\\ \frac{\partial \alpha^{\text{**}}}{\partial \beta }=-\frac{\delta^{2}\gamma \rho^{2}\tau^{2}}{{\left(\beta \gamma \delta^{2}+\rho^{2}\tau^{2}+1\right)}^{2}}\leq 0 \end{array}$$

The above analysis shows that the incentive coefficient α^**^, which introduces performance evaluation, is a decreasing function with respect to β, δ, and γ and an increasing function with respect to ρ and τ. Therefore, the maximum incentive coefficient set by the government is positively related to the ability of hospitals to share and use EHRs and the value-added coefficient of EHR, while it is negatively related to the investment cost coefficient of hospitals, the intensity of hospitals' risk avoidance, and the intensity of the risk posed by random perturbations.

Similarly, the partial derivatives of Eq. (14) for each parameter can be obtained:


$$\begin{array}{l} \frac{\partial k^{\text{**}}}{\partial \rho }=\frac{\tau \left(\beta \gamma \delta^{2}-\rho^{2}\tau^{2}+1\right)}{{\left(\beta \gamma \delta^{2}+\rho^{2}\tau^{2}+1\right)}^{2}}\geq 0\\ \frac{\partial k^{\text{**}}}{\partial \tau }=\frac{\rho \left(\beta \gamma \delta^{2}-\rho^{2}\tau^{2}+1\right)}{{\left(\beta \gamma \delta^{2}+\rho^{2}\tau^{2}+1\right)}^{2}}\geq 0\\ \frac{\partial k^{\text{**}}}{\partial \gamma }=-\frac{\beta \delta^{2}\rho \tau }{{\left(\beta \gamma \delta^{2}+\rho^{2}\tau^{2}+1\right)}^{2}}\leq 0\\ \frac{\partial k^{\text{**}}}{\partial \delta }=-\frac{2\beta \delta \gamma \rho \tau }{{\left(\beta \gamma \delta^{2}+\rho^{2}\tau^{2}+1\right)}^{2}}\leq 0\\ \frac{\partial k^{\text{**}}}{\partial \beta }=-\frac{\delta^{2}\gamma \rho \tau }{{\left(\beta \gamma \delta^{2}+\rho^{2}\tau^{2}+1\right)}^{2}}\leq 0 \end{array}$$

The above analysis shows that the performance evaluation effectiveness coefficient k^**^ is negatively related to β, δ and γ, and positively related to ρ and τ. The effectiveness coefficient of performance evaluation will increase with the ability of hospitals to share and use EHR, as well as the value-added coefficient of EHR. However, it will decrease with the increase of hospital investment cost coefficient, hospital risk avoidance intensity, and risk intensity caused by random disturbances.

### Simulation and comparative analysis of incentive model

To verify the effectiveness of the incentive model, MATLAB was used for numerical simulation to visually compare the incentive effects before and after the introduction of performance evaluation and the impact of each key factor on the incentive mechanism. If the incentive coefficients and benefit levels after the inclusion of performance evaluation were greater than the previous coefficients and levels, provided that β*γδ*^2^−(1−ρ^2^)τ^2^≥0 and β^2^γδ^2^−(1−β)ρ^3^τ^3^≥0 (Appendix A). Therefore, it is possible to β = 0.7, γ = 0.6, δ = 0.3, τ = 0.2, ρ = 0.8. When simulating the impact of a certain parameter on the incentive effect, the values of other parameters remain unchanged.

#### Impact of hospital investment cost coefficient β on incentive coefficients and benefit levels

**Figure 1:**
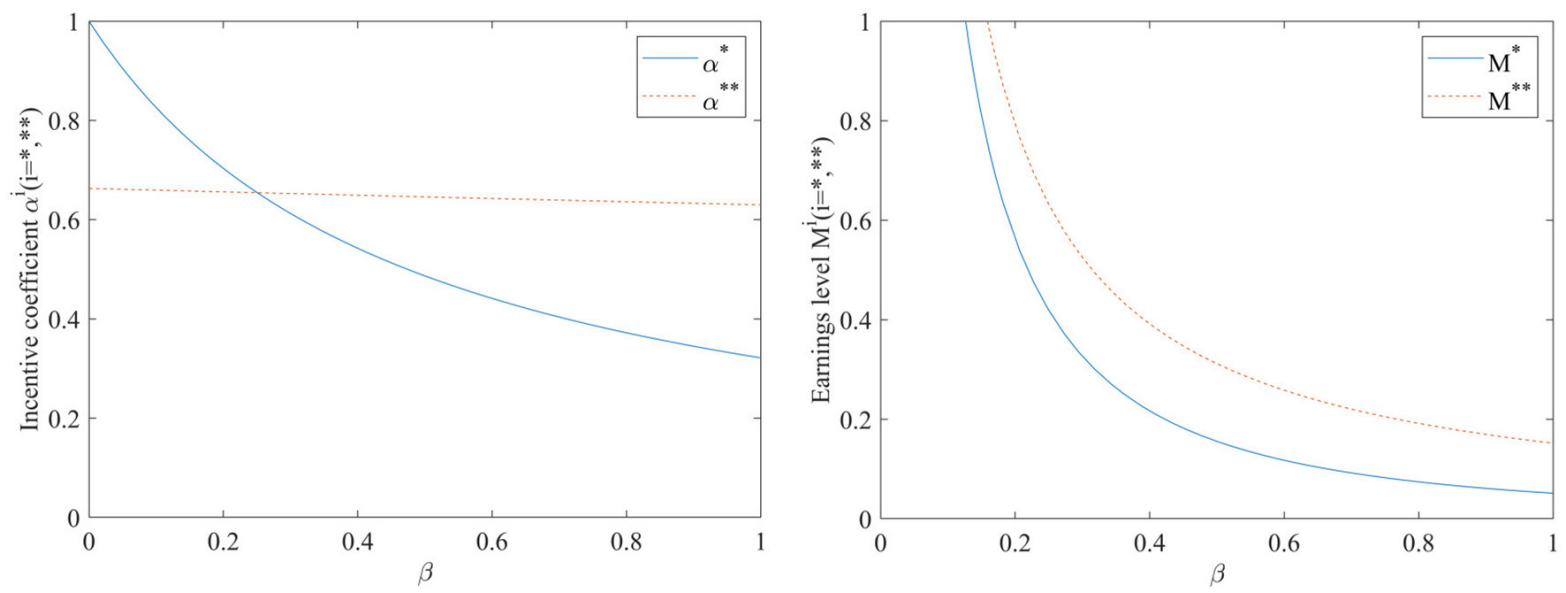
Impact of hospital investment cost coefficient β on incentive coefficients and benefit levels.

As shown in Figure 1, the hospital investment cost coefficient β has a similar effect on the benefit levels before and after the inclusion of the performance evaluation monitoring signal, both of which gradually decrease as the hospital investment cost coefficient β increases. However, the incentive coefficients are more sensitive to the hospital investment cost coefficient β, which is not added to the performance evaluation. In the absence of a performance evaluation supervisory signal, the incentive effect is very good if the hospital investment cost coefficient is very low, and the incentive coefficient decreases rapidly as the hospital investment cost coefficient increases; when the hospital investment cost coefficient increases to 1, i.e., when the cost of EHR integration is fully borne by the hospital, the incentive coefficient becomes 0, indicating that the incentive will be lost altogether. Meanwhile, Figure 1 also indicates that the benefit level after adding performance evaluation is consistently higher than that without adding performance evaluation.

#### Impact of hospital risk avoidance intensity γ on incentive coefficients and benefit levels

**Figure 2:**
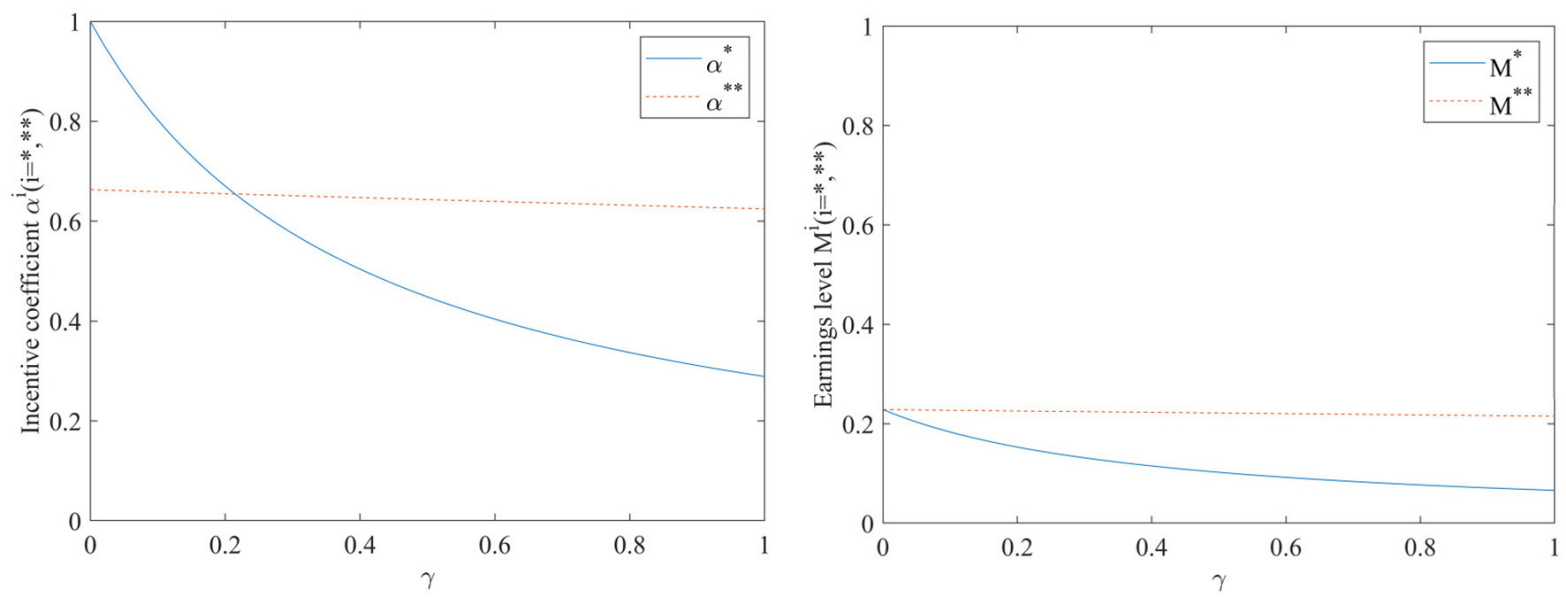
Impact of hospital risk avoidance intensity γ on incentive coefficients and benefit levels.

As can be seen from Figure 2, both the incentive coefficient and the benefit level decrease gradually with the increase of hospital risk avoidance intensity, regardless of the inclusion of performance evaluation supervisory signals or not. The incentive coefficient is more sensitive to the hospital risk aversion intensity γ. When the performance evaluation supervisory signal is added, the strength of the hospital's risk avoidance intensity γ on both the incentive coefficient and the benefit level becomes smaller, suggesting that conducting performance evaluation is greatly beneficial for stabilizing the incentive effect. At this point, the impact of hospital risk avoidance intensity γ on the benefit level is consistently larger than when no performance evaluation supervisory signals are included, which may be mainly due to the additional costs associated with conducting performance evaluations.

#### Impact of capacity to use EHR ρ on incentive coefficients and benefit levels

**Figure 3:**
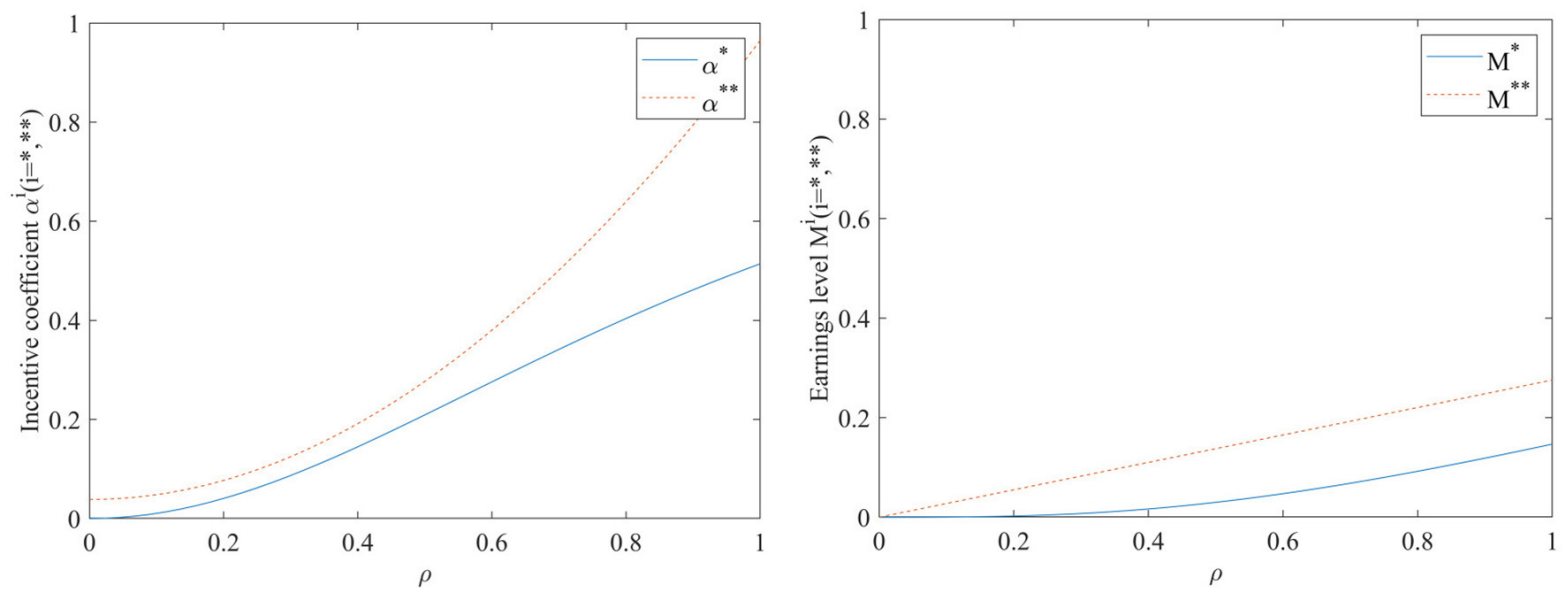
Impact of capacity to use HER ρ on incentive coefficients and benefit levels.

As can be seen in Figure 3, both the incentive coefficients and the hospital benefit level will increase as the ability to use hospital records ρ increases, regardless of whether a performance evaluation supervisory signal is included in the incentive model. The impact of the hospital's ability to use EHR before adding performance evaluation supervision signal on incentive coefficients and benefit levels is smaller than after adding supervision signal. Conversely, after adding performance evaluation supervision signal, the hospital's ability to use EHR has a higher impact on improving incentive effectiveness and earnings level. It indicates that conducting performance evaluation has a positive promoting effect on achieving sustainable sharing and use of EHR.

#### Impact of EHR value-added coefficient τ on incentive coefficients and benefit levels

**Figure 4:**
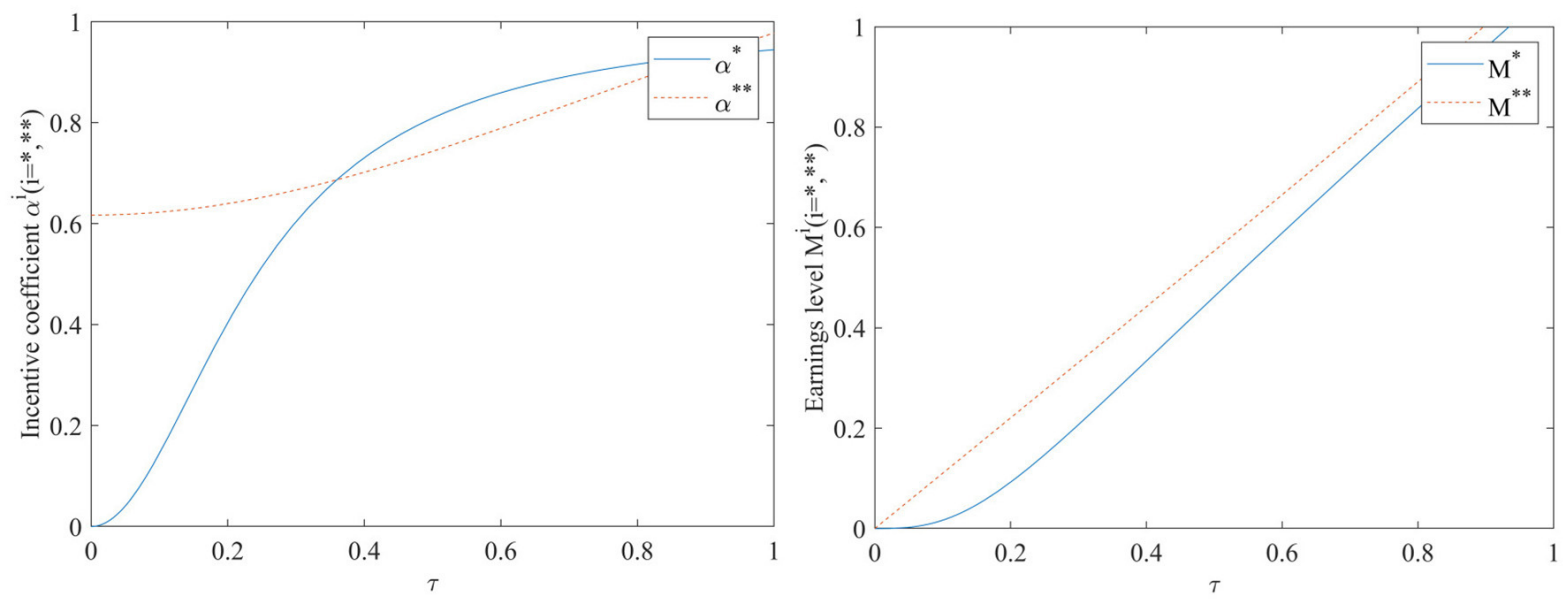
Impact of EHR value-added coefficient τ on incentive coefficients and benefit levels.

As can be seen in Figure 4, the incentive coefficient and earnings level increase with the increase of value-added coefficient, regardless of whether or not the performance evaluation supervision signal is included in the model. When a performance evaluation supervision signal is added to the model, the incentive coefficient is at a high level even when the value-added coefficient is close to 0, and there is an almost linear increasing relationship between the value-added coefficient τ and the earnings level. In the case of adding performance evaluation supervision signal, the impact of the value-added coefficient on the earnings level is always greater than that of not adding performance evaluation supervision signal.

#### Impact of exogenous random disturbance factors δ on incentive coefficients and benefit levels

**Figure 5:**
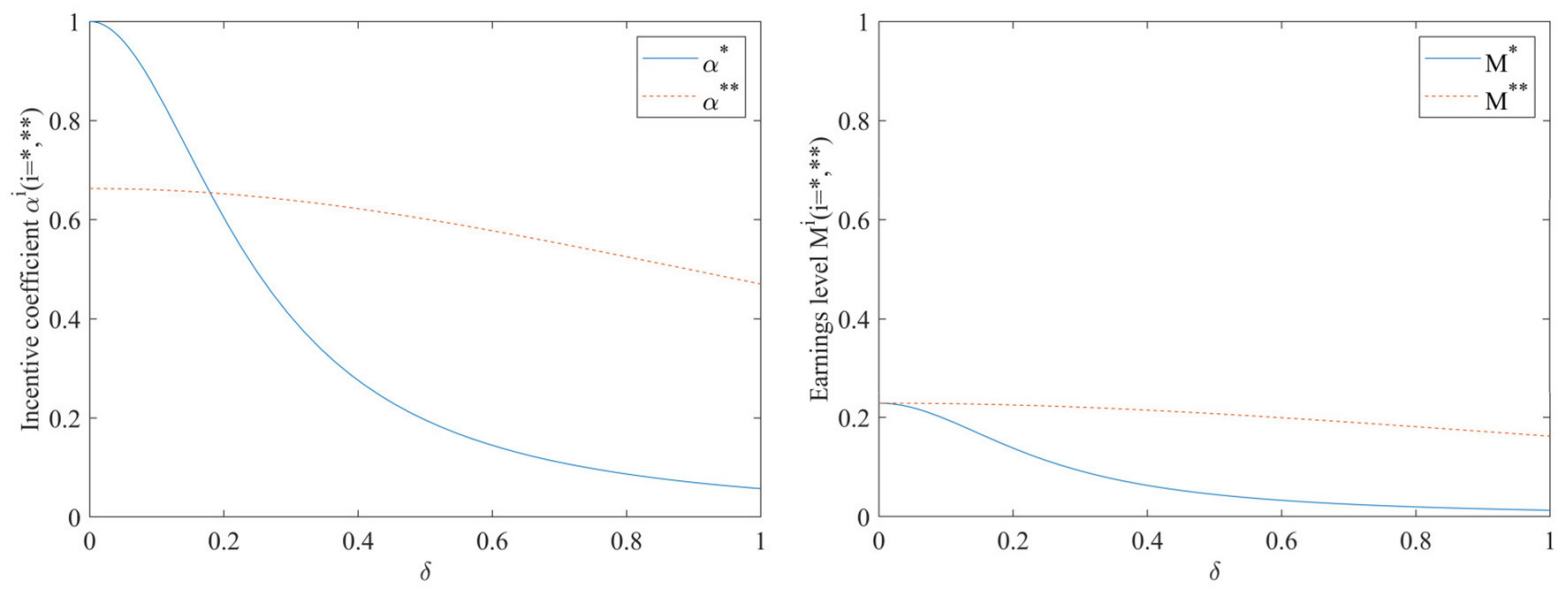
Impact of exogenous random disturbance factors δ on incentive coefficients and benefit levels.

As can be seen in Figure 5, both the incentive coefficients and the earnings levels decrease as the exogenous random disturbance factors δ increases. When the performance evaluation supervision signal is not added, the influence of exogenous random disturbance factors on the incentive coefficient is very significant. As the exogenous random disturbance factors increase, the incentive coefficient will significantly decrease; At this point, the earnings level is also more sensitive to the impact of exogenous random disturbances. When the performance evaluation supervision signal is added, the effect of the exogenous random disturbance factors δ on both the incentive coefficients and the earnings level will become smoother. Figure 5 also shows that the earnings level with the inclusion of the performance evaluation supervision signal is consistently higher than that in the case of non-inclusion.

#### The effect of β, γ, ρ, τ, and δ on the performance evaluation effectiveness

**Figure 6:**
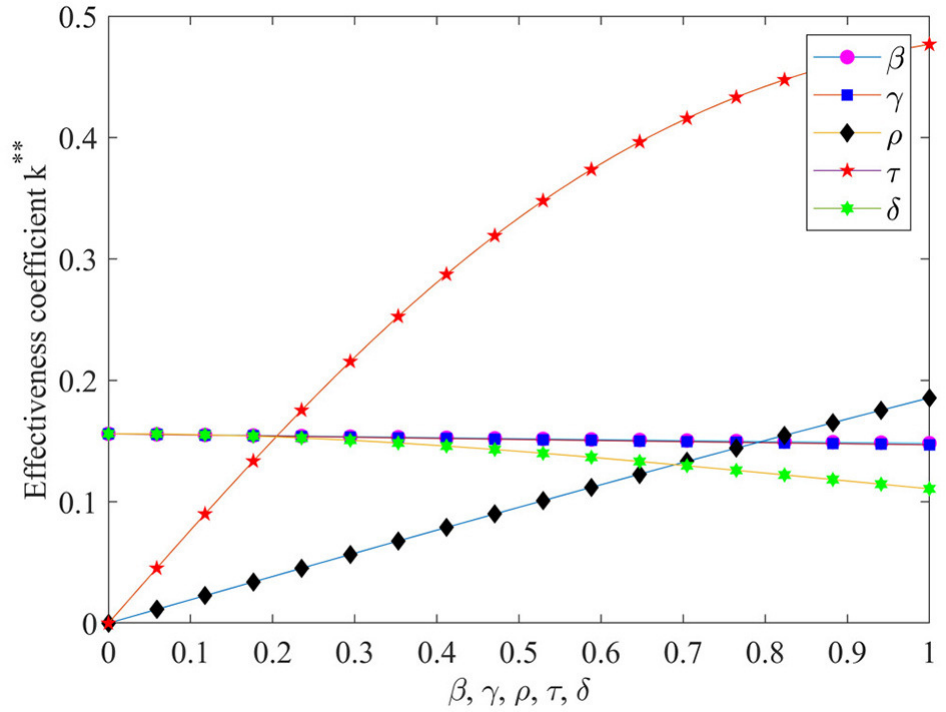
The effect of *β*, *γ*, *ρ*, τ and *δ* on the performance evaluation effectiveness. **It is used to distinguish between K *and K **.

To further analyze the effect of parameters such as β, γ, ρ, τ and δ on the effectiveness of performance evaluation, Figure 6 is plotted according to Eq. (14).

As can be seen from Figure 6, the value-added coefficient τ of the EHR has the greatest impact on performance evaluation effectiveness, and as the value-added coefficient increases performance evaluation effectiveness increases dramatically. The ability to use EHR ρ also has a significant positive effect on the effectiveness coefficient of performance evaluation. With the increase in the ability to use EHR the performance evaluation effectiveness coefficient also increases significantly. The effects of hospital investment cost coefficient β and hospital risk avoidance intensity γ on the performance evaluation effectiveness coefficient are basically the same, as they increase performance evaluation effectiveness coefficient slowly decreases. The effect of the exogenous random disturbance factors δ on the effectiveness coefficient of performance evaluation is slightly more pronounced than that of the intensity of risk avoidance γ, which also has a negative influence effect.

## Conclusions and insights

In this paper, on the basis of constructing the basic incentive model, performance evaluation was introduced as the metric basis and supervisory signal of the hospital's efforts, and used numerical simulation methods to verify the effectiveness of incentive mechanism design. The findings of this paper not only clarify the motivational elements and their role relationships in EHR integration, but also provide a sustainable incentive scheme for achieving the goal of EHR integration, which will be an important reference value for further optimizing related policies and improving the promotion mechanism. The findings of this paper and its managerial implications mainly include the following aspects.

Regardless of whether performance evaluation supervision signal is introduced or not, the incentive coefficient is an increasing function of the hospital's ability to use EHR and the EHR value-added coefficient, but a decreasing function of the hospital's investment cost, hospital risk avoidance intensity, and exogenous random disturbance factors. The ability to use EHRs of hospitals and the value-added coefficient of EHRs has a significant positive contribution to performance evaluation effectiveness. Therefore, seeking to enhance the capacity of hospitals to use EHR and to explore and expand the value-added space of EHR are 2 key paths to promote the sustainable sharing and use of EHR.

Neither the level of benefits nor the incentive coefficient for hospitals to share and use EHRs is related to the one-time government subsidy. The one-time government subsidy is a “hygiene factors” that is not a driving force for hospitals to share and use EHR for a long time, and cannot provide sustainable incentives. The government provides incentive subsidies based on the sharing and use performance of EHR, which is a more reasonable incentive method.

Conducting performance evaluation plays an important role in stabilizing the effectiveness of incentives. After incorporating the supervisory signal of performance evaluation into the model, the hospital's ability to use EHR has a higher impact on improving incentive effectiveness and earnings levels; The impact of exogenous random disturbance factors on incentive coefficients and earnings levels has also become more muted; The impact of the value-added coefficient on the earnings level is always greater than that without the addition of performance evaluation supervision signal. Therefore, effective performance evaluation is not only necessary but also important to advance the sustainable sharing and use of EHRs. The sharing and use of EHR should be included in the standardized maturity measurement programme for hospital information connectivity conducted annually in China to ensure objectivity and fairness in performance evaluation.

## Data availability statement

The original contributions presented in the study are included in the article/supplementary material, further inquiries can be directed to the corresponding author.

## Author contributions

SHT: Formal analysis, Visualization, Writing – original draft. RJ: Formal analysis, Funding acquisition, Writing – original draft. YC: Methodology, Supervision, Writing – review & editing.

## Appendix

### Appendix A

(1) Analysis of changes in incentive coefficients before and after the introduction of performance evaluation

The incentive coefficient for adding performance evaluation supervision signal is $\alpha^{**}=\frac{\rho^{2}+\tau^{2}}{\beta \gamma \delta^{2}+\rho^{2}\tau^{2}+1}$, The incentive coefficient without the inclusion of the performance evaluation supervision signal is $\alpha^{*}=\frac{\rho^{2}\tau^{2}}{\beta \gamma \delta^{2}+\rho^{2}\tau^{2}}$. Therefore, there is

$$\begin{array}{l} \;\alpha^{0}=\alpha^{**}-\alpha^{*}=\frac{\rho^{2}+\tau^{2}}{\beta \gamma \delta^{2}+\rho^{2}\tau^{2}+1}-\frac{\rho^{2}\tau^{2}}{\beta \gamma \delta^{2}+\;\rho^{2}\tau^{2}}\\ \;=\frac{\beta \gamma \delta^{2}-(1-\rho^{2})\tau^{2}}{\left(\beta \gamma \delta^{2}+\rho^{2}\tau^{2}+1)(\beta \gamma \delta^{2}+\rho^{2}\tau^{2}\right)} \end{array}$$

To make α^0^ ≥ 0 means that in order for performance evaluation to be effective, it is necessary to β*γδ*^2^ − (1 − *ρ*^2^)τ^2^ ≥ 0.

(2) Analysis of changes in the earnings level before and after the introduction of performance evaluation

The benefit level included in the performance evaluation supervision signal is $M^{**}=\frac{\rho^{3}\tau^{3}+\rho \tau }{\beta \left(\beta \gamma \delta^{2}+\rho^{2}\tau^{2}+1\right)}$, The benefit level without adding performance evaluation supervision signals is $M^{*}=\frac{\rho^{3}\tau^{3}}{\beta^{2}\gamma \delta^{2}+\beta \rho^{2}\tau^{2}}$. Therefore, it can be obtained that:

$$M^{0}\text{ =}M^{**}-M^{*}\text{ =}\frac{\beta^{2}\gamma \delta^{2}+(\beta -1)\rho^{3}\tau^{3}}{\beta (\beta \gamma \delta^{2}+\rho^{2}\tau^{2}+1)(\beta^{2}\gamma \delta^{2}+\beta \rho^{2}\tau^{2})}$$

To make *M*^0^ ≥ 0, which means that the earnings level after adding performance evaluation increases, it is necessary to meet the following requirements: *β*^2^*γδ*^2^ − (1 − *β*)*ρ*^3^τ^3^ ≥ 0.
